# The effects of creatine loading on cellular integrity and jump performance across the menstrual cycle

**DOI:** 10.1080/15502783.2025.2533651

**Published:** 2025-08-20

**Authors:** Sam R. Moore, Amanda G. Mutuwa, Ann Brown, Abbie E. Smith-Ryan

**Affiliations:** a Department of Exercise and Sport Science, University of North Carolina at Chapel Hill, Chapel Hill, NC, USA; b Department of Sport, Exercise and Rehabilitation, Northumbria University, Newcastle Upon Tyne, UK; c Department of Movement Sciences, University of Idaho, Moscow, ID, USA

**Keywords:** Creatine, menstrual cycle, bioelectrical impedance, athletic performance, dietary supplements

## Abstract

**Background:**

Creatine monohydrate (CrM) supplementation may offer unique benefits to active women through augmented cellular fluid outcomes. This study sought to evaluate the effects of CrM supplementation on whole-body 50 kHz phase angle (PhA; °) and countermovement jump height (CMJ; cm) across the menstrual cycle (MC).

**Methods:**

Twenty moderately active females (mean±standard deviation: Age: 25.6±5.6 yrs; Body mass: 66.2±7.0 kg; %fat: 25.7±6.8%) were randomized to a 4×5g/day for 5 days of either CrM (n=10) or non-caloric placebo (PL; n=10), as well as randomized to MC start phase (follicular [FP] or luteal phase [LP]). PhA, measured via bioelectrical impedance analysis (Inbody 770, BioSpace, Seoul, Republic of Korea), and CMJ, evaluated using a jump mat (Just Jump Mat, Probotics Inc., Huntsville, AL), were measured at pre- and post-supplementation timepoints in the FP and LP. Acute hydration was measured prior to all testing, using urine specific gravity (USG), to ensure adequate hydration status. Repeated measures analysis of covariance tests were used to assess pre- and post-supplementation differences between groups (CrM, PL) in PhA and CMJ, covaried for USG and lean mass, respectively.

**Results:**

The CrM group demonstrated significantly greater PhA at the LP post timepoint (mean difference [PL–Cr]±standard error: –0.37±0.17°; p=0.05) when compared to PL. The CrM group also demonstrated a significant change from pre to post timepoints in the FP (pre-post: -0.18±0.08°; p=0.05), while PL saw no significant change (–0.08±0.08°; p=0.35). CMJ results demonstrated a significant decrease between phases at the post timepoint for the PL group (FP–LP: 3.82±1.54 cm; p=0.02), despite no differences across time or phase in the CrM group (p>0.05).

**Conclusions:**

Taken together, CrM supplementation supported improved cellular health, as indicated through raw bioimpedance measures such as PhA. Such improvements may help maintain physical performance across the MC, particularly in the mid to late LP when approaching menstruation.

## COI/Disclosures

The creatine monohydrate used in this study was donated free of charge from AlzChem Trostberg GmbH (Trostberg, Germany), for which A.E.S.-R. serves as a scientific advisor.

